# Budget line items for immunization in 33 African countries

**DOI:** 10.1093/heapol/czaa040

**Published:** 2020-05-27

**Authors:** Ulla K Griffiths, Jennifer Asman, Alex Adjagba, Marina Yo, James O Oguta, Chloe Cho

**Affiliations:** c1 Health Section, UNICEF, 3 UN Plaza, New York, NY 10017, USA; c2 Social Policy Section, UNICEF, 3 UN Plaza, New York, NY 10017, USA; c3 Health Section, UNICEF, Eastern and Southern Africa Regional Office, PO Box 44145, Nairobi 00100, Kenya; c4 Health Section, UNICEF, Western and Central Africa Regional Office, P.O. Box 29720, Yoff Dakar, Senegal; c5 International Budget Partnership, 750 First Street NE, Suite 700, Washington, DC 20002, USA

**Keywords:** Budget, expenditure, vaccine, immunization, public financial management

## Abstract

When seeking to ensure financial sustainability of a health programme, existence of a line item in the Ministry of Health (MOH) budget is often seen as an essential, first step. We used immunization as a reference point for cross-country comparison of budgeting methods in Sub-Saharan African countries. Study objectives were to (1) verify the number and types of budget line items for immunization services, (2) compare budget execution with budgeted amounts and (3) compare values with annual immunization expenditures reported to WHO and UNICEF. MOH budgets for 2016 and/or 2017 were obtained from 33 countries. Despite repeated attempts, budgets could not be retrieved from five countries (Chad, Eritrea, Guinea Bissau, Somalia and South Sudan), and we were only able to gather budget execution from eight countries. The number of immunization line items ranged between 0 and 42, with a median of eight. Immunization donor funding was included in 10 budgets. Differences between budgeted amounts and expenditures reported to WHO and UNICEF were greater than 50% in 66% of countries. Immunization budgets per child in the birth cohort ranged from US$1.37 (Democratic Republic of Congo) to US$67.51 (Central African Republic), with an average of US$10.05. Out of the total Government health budget, immunization comprised between 0.04% (Madagascar) and 5.67% (Benin), with an average of 1.98% across the countries, when excluding on-budget donor funds. It was challenging to obtain MOH budgets in many countries and it was largely impossible to access budget execution reports, preventing us from assessing budget credibility. Large differences between budgets and expenditures reported to WHO and UNICEF are likely due to inconsistent interpretations of reporting requirements, diverse approaches to reporting donor funds, challenges in extracting the relevant information from public financial management systems and broader issues of public financial management capacity in MOH staff.

**KEY MESSAGES**

- Not all countries have a line item for vaccines or immunization in their budget. Transition from input based to programme budgeting can lead to removal of immunization line items.
- Budget execution reports are often not publicly available. Our limited sample of execution reports suggests low budget execution of vaccine procurement in some countries.
- Budget structure and number of line items for immunization vary substantially between countries.

## Introduction

The national budget is a document that, once approved by the legislature, authorizes the government to raise revenues, incur debts and make expenditures in order to achieve certain goals (Norton and Elson, 2002). It reflects the priorities given to different institutions and purposes and is the key instrument for translating government policies into action. When seeking to ensure financial sustainability of a specific health intervention, existence of a budget line in the Ministry of Health (MOH) budget is often seen as an essential, first step. A line item increases visibility of government support in budget prioritization, allocation and execution. A separate budget line may make it more likely that budget decision-makers explicitly allocate resources for immunization.

In practice, the budget line may take a range of forms. In a traditional input-based budget, expenditure is typically structured according to administrative units and types of expenses, with specification of inputs, such as personnel, supplies, utilities and equipment (Rajan *et al.*, 2016). For example, the MOH budget may set out an administrative structure, such as the Directorate of Immunization Services, and include separate line items for different inputs or categories of inputs. This approach, the norm in many countries, aims to achieve transparency and accountability, but it has been criticized for restricting flexibility and leading to inefficient resource allocation (Rajan *et al.*, 2016). Increasingly, countries are moving to performance budgeting using a programme structure (Robinson, 2014). The aim is to strengthen the links between funding and results by focusing on services delivered rather than inputs purchased (Radisic *et al.*, 2016). A programme budget structure allocates funds to programmes, which are established based on defined sets of services that deliver the core functions of a ministry. In this context, immunization could be a programme, a subprogramme or activity within the MOH budget, depending on how the programme structure is defined.

This study is a cross-country comparison on how immunization is reflected in MOH budgets in Sub-Saharan African countries. Since vaccine supply is both a basic commodity and a high-value item, it can be expected to be given a specific line item in MOH budgets. Study objectives were to (1) compare the number and types of budget line items for immunization services, (2) analyse budgeted amounts and compare these with amounts executed and (3) compare budgeted and executed values with annual immunization expenditures reported to WHO and UNICEF.

## Methods

### Data collection

The study included 38 countries eligible for support from Gavi, the Vaccine Alliance. This included 21 countries in UNICEF’s West and Central Africa region and 17 countries of the East and Southern Africa region. UNICEF country offices were asked to obtain a copy of the relevant pages in the 2016 and 2017 MOH budgets and budget execution reports that showed immunization allocations and funds expended. The in-country requests for budget documents were complemented by a search for MOH budgets on government websites and two online data portals: the World Bank’s Open Budget Portal (BOOST) and the Collaborative Africa Budget Reform Initiative (CABRI) (CABRI, 2019; World Bank, 2019).

### Data analysis

#### Number and types of line items

The number of immunization line items were counted and categorized according to most common types. Since 1998, the WHO and UNICEF have jointly collected information on immunization through a standard questionnaire sent to all Member States once a year (World Health Organization, 2019b). This ‘Joint Reporting Form’ (JRF) has asked if ‘there is a line item in the national budget for the purchase of vaccines used in routine immunization’ (World Health Organization, 2019b) since 1998. We verified country responses by reviewing published MOH budgets for such a line item.

#### Amounts budgeted for immunization

Budget values were converted to US dollars using exchange rates from the International Monetary Fund (2019). The proportion of the total government health budget assigned to immunization was calculated. The immunization budget per child in the birth cohort was estimated using demographic data from the United Nations Population Division (2019). The difference between the immunization and the vaccine budget per child in the birth cohort was calculated to determine amounts budgeted for vaccine delivery, such as transport, communication materials, etc. Budget execution rates were calculated for vaccines and immunization for those countries with expenditure data available.

The number of budget line items was correlated with the size of the immunization budget per child in the birth cohort, and Spearman rank correlation coefficients were calculated to assess if there was significant correlation between the two variables, using Stata version 16.

In the JRF, the following questions are asked as part of ‘Financing data’ (World Health Organization, 2019b):

- What is the government expenditure on vaccines used in routine immunization?
- What is the government expenditure on routine immunization, including vaccines?

We compared budget and budget execution values with JRF reported expenditures. We aligned our data with the JRF definition of government expenditure, which includes on-budget financing from donors (World Health Organization and UNICEF, 2015). JRF definitions are included in Supplementary Annex S1.

Gavi (2020) offers financial support to countries for 17 different vaccines, primarily new and underused vaccines, including pneumococcal conjugate and rotavirus vaccines. Gavi does not support ‘traditional’ vaccines, such as Bacille Calmette Guerin and oral polio vaccines. Countries receiving Gavi vaccine support are obliged to co-finance a fraction of the vaccine costs for routine immunization by co-procuring a portion of vaccines (Henderson *et al.*, 2016). All study countries used UNICEF as their procurement agent and fulfilled co-financing obligations by transferring the required amount to UNICEF. UNICEF records of funds received for co-financing vaccine purchases were compared with the amount countries had included for vaccine purchases in their budget.

#### Inclusion of donor funding

According to the 2005 Paris declaration on aid effectiveness, aid flows should be recorded in country budgets (OECD, 2008). Donors have committed to ‘providing timely, transparent and comprehensive information on aid flows so as to enable partner authorities to present comprehensive budget reports to their legislatures and citizens’ (OECD, 2008). We examined MOH budget documents for on-budget donor funding for immunization. This included an electronic search for the word ‘Gavi’ in the full budget document.

#### Budget classification structure

MOH budgets were categorized as either using a programmatic or an input-based (administrative) structure. Existence of an immunization line item, and the number and types of line items were compared across the two categories.

## Results

### Availability of budget information

Several budget document types were obtained, including budget laws, budget estimates, medium-term expenditure frameworks and execution reports (see references in Supplementary Annex S2). MOH level data were harder to obtain than overall national budget information, but national budgets did not include enough detail on MOH allocations to identify immunization. MOH budgets from either 2016, 2017 or both years were gathered from 32 countries and the Burkina Faso budget, which was only available through BOOST, for 2014 and 2015 (Table 1). Hence, a total of 33 countries were included in the analysis. MOH budgets from Chad, Eritrea, Guinea Bissau, Somalia and South Sudan could not be accessed despite repeated attempts. Budget execution data could only be obtained from 8 of the 33 countries (24%); Burkina Faso, Cote d’Ivoire, Liberia, Mali, Mozambique, Niger, Rwanda and Senegal. Nineteen (58%) of the MOH budgets were available from at least one online source (Table 1). Nine were included in BOOST, eight on Government websites and seven in CABRI. The Ugandan and Kenyan MOH budgets were found in all three places.

**Table 1:** Number of immunization line items in Ministry of Health budget

Country	Online source for MOH budget	Number of line items 2016	Number of line items 2017	Budget execution available	Donor funding incorporated in budget	Budget structure
Angola	NA	5	5	No	No	Programme
Benin	NA	8	8	No	No	Input based
Burkina Faso^a^	BOOST	29	29	Yes	No	Input based
Burundi	Gov. website, BOOST	3	3	No	No	Input based
Cameroon	NA	NA	13	No	No	Programme
CAR	CABRI	16	16	No	Yes	Input based
Comoros	Gov. website	1	NA	No	No	Input based
Congo	NA	5	5	No	Yes	Input based
Côte d'Ivoire	NA	30	31	Yes	Yes	Input based
DRC	NA	8	8	No	No	Input based
Ethiopia	NA	NA	9	No	Yes	Input based
Gambia	NA	1	6	No	No	Input based
Ghana	Gov. website	3	0	No	No	Programme
Guinea	NA	14	17	No	No	Input based
Kenya	BOOST, CABRI, Gov. website	11	10	No	Yes	Input based
Lesotho	NA	6	11	No	No	Input based
Liberia	CABRI	2	2	Yes	No	Input based
Madagascar	NA	17	42	No	Yes	Input based
Malawi	Gov. website	0	0	No	No	Programme
Mali	BOOST	6	6	Yes	No	Input based
Mauritania	BOOST	6	10	No	No	Input based
Mozambique	NA	25	30	Yes	Yes	Input based
Niger	Gov. website, BOOST	10	10	Yes	No	Input based
Nigeria	Gov. website	6	9	No	NA	Input based
Rwanda	CABRI	14	8	Yes	Yes	Programme
Sao Tome	CABRI	1	1	No	No	Input based
Senegal	BOOST	13	14	Yes	Yes	Input based
Sierra Leone	Gov. website	1	1	No	No	Input based
Tanzania	NA	0	0	No	No	Programme
Togo	BOOST	2	2	No	No	Input based
Uganda	BOOST, CABRI, Gov. website	17	17	No	Yes	Programme
Zambia	CABRI	4	4	No	No	Input based
Zimbabwe	NA	NA	0	No	No	Programme

a Budgets for Burkina Faso are for 2014 and 2015.

CAR, Central African Republic; DRC, Democratic Republic of Congo; NA, not available.

### Existence of line item for vaccine procurement

All but one of the 33 countries responded ‘yes’ to the JRF question about whether they had a budget line item for purchase of vaccines. While Burkina Faso reported ‘yes’ in the years before 2015, the response was ‘no’ in 2015, 2016 and 2017 (World Health Organization, 2019a). However, we found that the Burkina Faso 2015 MOH budget did have a line item for vaccines, with US$1.7 million budgeted in both 2014 and 2015. The line item was within ‘Direction Prévention—Vaccination’ and called ‘Produits, vaccins médicaux’ (World Bank, 2020).

Of the 32 countries that responded ‘yes’, four did not have any line items related to immunization (Ghana, Malawi, Tanzania and Zimbabwe). Of the remaining 28 countries, 13 included a clear and specific line item for vaccines, with titles such as ‘vaccines’ or ‘vaccines and vaccination supplies’. A further five countries included vaccine-related line items, which referred to both immunization and non-immunization activities, with titles such as ‘vaccine and deworming supplies’, ‘purchase of pharmaceuticals, medical and veterinary’ or ‘medical and agricultural supplies’. For the purposes of this study, given the line items were listed within the budget for an organizational structure responsible for immunization, these five countries were accepted as having a vaccine line item [Central African Republic (CAR), Cote d’Ivoire, Mauritania, Togo and Uganda].

A further 10 countries had unspecific line items, such as ‘immunization programme’, ‘health and hygiene’ and ‘current transfers to other governments’ (Angola, Benin, Cameroon, Comoros, Madagascar, Mozambique, Rwanda, Sao Tome and Principe, Senegal and Sierra Leone). While the large value associated with some of these line items made it probable that it covered vaccine supplies, these were considered insufficiently clear. Based on this classification, 19 of the 33 (58%) countries were assessed as meeting the criteria of a budget line item for the purchase of vaccines.

The four countries that did not include any budget line items for immunization all used a programme budget structure. Ghana transitioned to a programme budget in 2014. While a line item called ‘National Vaccination Exercise’ was included in three of the subprogrammes in the 2016 MOH budget (population-based services, regional and district health services, and specialized health services), the amounts budgeted were too small to include vaccine procurement (Republic of Ghana, Ministry of Health, 2015). When the budget was restructured in 2017, there were no line items for immunization in the MOH budget. In 2016, the Ghana MOH decided to start financing vaccines using an allocation from the National Health Insurance Scheme (NHIS) which does not make its budget publicly available.

The Malawian Government transitioned to programme budgeting in 2016/17 (UNICEF, 2019). One of the performance measures for the Primary Health Care programme is ‘percentage of 1 year-old children fully immunized’ (Malawi Ministry of Finance Economic Planning and Development, 2016). There is, however, no line item for vaccines or immunization services in the Primary Health Care budget. A line item for ‘Medical supplies and expenses’ exists for all six MOH programmes, but no funds were budgeted in ‘Secondary Health Care’ and only small amounts in ‘Primary Health Care’ and ‘Management and Administration’. The programmes ‘Support to Service Delivery’, ‘National Level Programs’ and ‘Tertiary Health Care’ were allocated 51%, 38% and 10% of the medical supplies budget, respectively. It is possible that vaccines are covered in ‘National Level Programs’. The Tanzania Ministry of Health and Social Welfare budget is structured according to five programmes; ‘Administration’, ‘Curative Services Delivery’, ‘Preventive Services Delivery’, ‘Food and Drug Control’ and ‘Health Training’. Curative and Preventive Services Delivery each have a budget line for ‘medical supplies and services’, comprising 43% of the preventive budget and 1% of the curative budget. It is likely that vaccines are included in Preventive Services Delivery.

The Zimbabwe budget has three programmes: ‘Policy and Administration’, ‘Public Health’ and ‘Primary Health Care and Hospital Care’. The Public Health subprogramme ‘family health’ has a performance indicator for ‘coverage for vaccine preventable conditions’. However, the family health budget is a lump sum with no line items. The Public Health programme has a line item for ‘goods and services’ with US$283 000 budgeted in 2016 and a dramatic increase to US$47 million in 2017 (Ministry of Finance and Economic Development, 2016).

#### Number of line items

The number of immunization line items varied widely, ranging from 0 in Ghana (2017), Malawi, Tanzania and Zimbabwe to 42 in Madagascar’s 2017 budget (Table 1). Five countries used a programme budget structure and still had immunization line items; Angola, Cameroon, Ghana (2016), Rwanda and Uganda. The average number of immunization line items in these countries were 9.8 in 2016 and 11.0 in 2017. Countries with input-based budgets had an average of 9.2 line items in 2016 and 11.6 in 2017.

The number of line items changed in 12 of the 29 countries with budgets available for 2 years (Table 1). Madagascar increased the number of line items for ‘Vaccination Services’ from 17 in 2016 to 42 in 2017. The country introduced a new budgeting structure in 2017 that added three additional programmes for immunization; ‘Support for the Expanded Programme of Vaccination’, ‘Surveillance for Vaccine Preventable Diseases’ and ‘Technical Services for Vaccination’ (Repoblikan’i Madagasikara, 2015, 2016). Rwanda was the only country with a notable decrease in the number of line items between the 2 years; 14 items in 2016 and 8 in 2017. This was due to removal of six line items within the Vaccine Preventable Disease budget: ‘Water and Energy’, ‘Rental Costs’, ‘Insurances and Licenses’, ‘Professional and Contractual Services’, ‘Maintenance and Repairs’ and ‘Acquisition of Other Machinery and Equipment’ (Republic of Rwanda, 2017).

### Types of line items

Several countries included line items to reflect operational activities for delivery of immunization services, such as office supplies or stationery (16 countries, 55% of countries with immunization budgets), and fuel or transport (14 countries, 48%) (Table 2). Eleven countries (38%) included line items for maintenance of equipment, office buildings or cars. Only a few countries included line items for activities essential to vaccine delivery. Two countries had a budget line for cold chain (Niger and Zambia, 7%), five countries including vaccination campaigns (17%) and three countries had surveillance (CAR, Congo and Madagascar). Gavi vaccine co-financing had a specific line item in five countries: Burundi, Cameroon, Cote d’Ivoire, DRC and Niger. Angola and Mozambique included immunization line items in their provincial budgets.

**Table 2:** Number and types of line items in immunization budgets (most recent year available)

Country	Vaccine supplies	Gavi co-financing	Staff/salaries/ allowances	Office supplies	Fuel and transport	Utilities	Maintenance	Vaccination campaigns	Printing/ Child Health Records	Surveillance	Cold chain	Other	Total
Angola												5	5
Benin			7									1	8
Burkina Faso	1		12	1	3		2		1			9	29
Burundi	1	1										1	3
Cameroon		2	1	1	2		2		1			4	13
CAR	1		1	1	3			3	1	1		5	16
Comoros												1	1
Congo	1							2		1		1	5
Côte d'Ivoire	2	1	4	4	3	2	7					8	31
DRC	1	1	1	1	1							3	8
Ethiopia	1		1	1	1				1			4	9
Gambia	1			2			1					2	6
Guinea	2			2	2	1	6					4	17
Kenya	2			1	2	1						4	10
Lesotho	1		3	1					2			4	11
Liberia	2											0	2
Madagascar			3	7	9	9	4		1	1		8	42
Mali	1		1		2							2	6
Mauritania	1			2			4					2	9
Mozambique												30	30
Niger	3	1		2	1			1			1	1	10
Nigeria	3											6	9
Rwanda				1	2	1						4	8
Sao Tome												1	1
Senegal					3		1					10	14
Sierra Leone												1	1
Togo	1											1	2
Uganda	1		1	3	3	1	2					6	17
Zambia	1								1		1	1	4
Total	27	6	35	30	37	15	29	6	8	3	2	129	327

### Budgeted amounts

Given the limited available execution data, comparisons were first made between vaccine budget allocations and vaccine expenditures reported in the JRF. While comparing allocations with expenditure may lead to discrepancies if budget execution is poor, this provides an indicative comparison of the consistency between the two data sources.

For Government expenditures on vaccines, the comparison revealed >50% differences in 16 of the 19 countries with a vaccine procurement line item, with variation in both directions (Table 3). Since 13 of these countries reported lower expenditure in the JRF response than their budget allocation, the disparities could be due to poor budget execution. The largest monetary differences were in Ethiopia and Nigeria. Ethiopia’s vaccine budget amounted to US$83.7 million in 2017, with on-budget Gavi support comprising 99% of the budget, but only US$12.2 million was reported to the JRF. Excluding donor support does not resolve the variation, with only US$0.75 million in government funds allocated to vaccines, less than the JRF reported expenditure. Nigeria’s vaccination budget for 2016 was US$4.9 million, but US$120 million were reported to the JRF. An explanation for this deviation could be that Nigeria used a World Bank IDA credit for vaccine procurement, which is not included in the budget line for vaccines (Wonodi and Adewumi, 2018). The only countries with <20% deviation between the budget and JRF values were Cote d’Ivoire and Congo (2017), Kenya (2017) and Uganda (2017). Removing Nigeria and Ethiopia, the average difference between budgets and JRF values was 66% across the 2 years.

**Table 3:** Vaccine budgets compared with vaccine expenditures reported in Joint Reporting Form and co-financing amounts (US$) (Figures in brackets exclude on-budget donor funding)

Country	2016 vaccine supply budget	2017 vaccine supply budget	2016 JFR Government vaccine expenditures	2017 JFR Government vaccine expenditures	2016 vaccine budget as % of JRF	2017 vaccine budget as % of JRF	2016 co-financing	2017 co-financing	2016 co-financing as % of vaccine budget	2017 co-financing as % of vaccine budget
Burkina Faso^a^	1 690 761	1 690 761	3 388 963	2 777 176	38	28	1 242 661	1 435 032	73	85
Burundi	707 129	695 042	510 053	1 003 776	139	69	511 140	494 940	72	71
CAR	455 306	489 611	124 244	115 961	366	422	167 326	121 950	37	25
Congo	4 974 636	515 380	1 047 203	419 176	475	123	488 566	746 760	10	145
Côte d'Ivoire	7 235 725 (3 746 604)	10 070 447(6 515 910)	7 423 912	8 069 068	97 (50)	125 (81)	1 334 440	916 555	18 (36)	9 (14)
DRC	5 983 990	2 193 001	3 775 045	NA	159	NA	10 039 985	0	168	0
Ethiopia	NA	83 703 491 (750 062)	11 835 511	12 190 577	NA	687 (6)	4 402 641	4 490 322	NA	5 (599)
Gambia	546 888	514 938	882 000	884 500	62	58	136 997	111 181	25	22
Guinea	2 862 661	2 208 705	328 920	194 511	870	1136	223 469	232 500	8	11
Kenya^b^	32 540 471 (6 925 810)	6 925 810	7 824 000	7 824 000	416 (89)	89	2 928 157	592 517	9 (42)	9
Lesotho	163 159	124 667	456 019	352 071	36	35	31 583	75 818	19	61
Liberia	650 000	477 404	296 050	180 500	220	264	296 050	194 637	46	41
Mali	5 690 525	5 625 423	3 180 350	4 261 159	179	132	1 677 712	1 750 795	29	31
Mauritania	397 309	838 120	601 825	550 325	66	152	303 887	199 617	76	24
Niger	5 171 129	4 567 165	3 534 761	2 696 731	146	169	0	0	0	0
Nigeria	4 883 658	25 184 626	120 000 000	35 480 412	4	71	29 268 750	38 100 622	599	151
Togo	1 011 790	1 030 760	445 686	561 763	227	183	448 855	446 197	44	43
Uganda	2 339 114	5 847 786 (2 923 893)	4 432 069	5 151 336	53	114 (57)	2 054 296	1 708 614	88	29 (58)
Zambia	3 712 603	4 100 461	1 946 125	2 000 617	191	205	1 678 997	1 639 856	45	40

Source of co-financing data is UNICEF Supply Division.

^a^ Burkina Faso values are for 2014 and 2015.

^b^ Kenya’s financial year is June–July. Co-financing is aligned with the financial year. 2016 values cover July 2016–June 2017. 2017 values cover July 2017- June 2018.

NA, not available; CAR, Central African Republic; DRC, Democratic Republic of Congo.

Vaccine budget allocations were generally coherent with records of co-financing amounts transferred to UNICEF for Gavi co-financing procurement, with co-financing being less than the vaccine budget (Table 3). Niger defaulted on co-financing payments in both 2016 and 2017 while DRC did so in 2016. Co-financing amounts exceeded the allocated vaccine budget in Congo (2017), DRC and Nigeria. When removing these countries as well as the countries that defaulted, co-financing comprised on average 37% and 30% of the vaccine budget in 2016 and 2017, respectively.

Comparison between immunization allocations in government budgets and JRF reported immunization expenditures showed differences exceeding 50% in 19 of 28 countries (JRF data were not available for Angola) (Table 4). Across the 2 years, the average difference was 331%. There was good alignment only in Benin (2017), Cameroon (2017), Cote d’Ivoire, Madagascar (2016), Mozambique (2017) and Sierra Leone (2017). Of the 19 countries with differences exceeding 50%, 14 (74%) had higher JRF expenditure values than the amounts included in the national budget, indicating that the discrepancies cannot be explained by low budget execution rates. When excluding on-budget donor funding, the differences were less in CAR, Congo Republic, Kenya, Madagascar 2017, Rwanda and Senegal. The contrary was the case for Madagascar 2016, which aligned well with the JRF data when on-budget donor funding was included. In Ethiopia, the differences were substantive both with and without donor funding, but in opposite directions.

**Table 4:** Government immunization budgets compared with immunization expenditures reported in the Joint Reporting Form (Figures in brackets exclude on-budget donor funding)

Country	2016 immunization budget	2017 immunization budget	2016 JFR Government immunization expenditures	2017 JFR Government immunization expenditures	Imm. budget as % of 2016 JRF x	Imm. budget as % of 2017 JRF	2016 imm. budget % of total Government health budget	2017 imm. budget % of total Government health budget
Angola	13 187 664	21 790 617	NA	NA	NA	NA	0.71	3.85
Benin	2 554 617	2 599 609	3 951 102	2 653 336	65	98	5.67	3.02
Burkina Faso	2 207 123	2 169 113	3 527 388	3 864 493	34	24	0.72	0.51
Burundi	712 568	700 481	597 351	1 208 865	119	58	1.19	0.74
Cameroon	NA	5 625 765	4 542 974	5 473 014	NA	103	NA	1.57
CAR	11 198 011 (1 606 013)	3 895 104 (1 370 911)	124 244	168 359	9013 (1293)	2314 (814)	19.52 (2.80)	8.97 (3.16)
Comoros	44 968	NA	72 289	90 820	62	0	1.26	NA
Congo	5 345 205	2 853 255 (1 963 365)	1 161 872	453 535	460	629 (433)	1.71	1.25 (0.86)
Côte d'Ivoire	8 307 265(4 818 144)	11 162 078 (7 607 540)	10 130 041	10 022 706	82	111	2.31 (1.34)	1.51 (1.03)
DRC	13 484 026	4 673 444	3 934 332	633 902	343	737	2.14	1.42
Ethiopia	NA	98 557 551 (9 589 559)	49 721 033	51 212 664	NA	192 (19)	NA	9.61 (0.94)
Gambia	546 888	530 429	883 400	886 600	62	60	NA	3.27
Guinea	2 886 757	5 130 062	497 671	1 506 409	580	341	3.31	3.50
Kenya	32 738 555 (7 123 894)	7 149 840	8 094 991	NA	404 (88)	NA	5.51 (1.20)	1.20
Lesotho	883 776	883 776	784 917	518 841	113	170	0.66	0.57
Liberia	650 000	477 404	758 936	858 500	86	56	0.84	0.62
Madagascar	1 302 628 (43 396)	29 594 519 (3 268 698)	1 250 038	2 467 250	104 (3)	119 (32)	1.31 (0.04)	25.56 (2.82)
Mali	6 036 313	5 942 526	16 984 040	7 405 244	36	80	3.03	2.46
Mauritania	468 467	910 963	1 089 278	1 857 831	43	49	0.55	0.93
Mozambique	2 394 196(2 012 791)	6 801 072(0)	5 851 418	6 062 517	41 (34)	112 (0)	0.90 (0.75)	2.11 (0)
Niger	6 581 286	5 898 754	4 188 796	4 236 769	157	139	4.42	4.54
Nigeria	50 821 961	41 439 457	185 000 000	78 145 947	27	53	5.15	4.17
Rwanda	4 019 255 (1 662 740)	3 442 586 (1 916 460)	1 618 699	1 797 713	248 (103)	191 (107)	2.81 (1.16)	2.78 (1.55)
Sao Tome	27 992	27 619	554 542	360 072	5	8	0.12	0.34
Senegal	8 881 861 (4 516 633)	6 304 005	3 158 583	10 176 155	281 (143)	62	3.51 (1.78)	2.24
Sierra Leone	608 639	518 428	430 985	494 492	141	105	2.39	2.95
Togo	1 180 422	1 116 657	4 389 546	4 798 970	27	23	1.08	NA
Uganda	25 741 952 (2 590 569)	27 220 225 (3 176 810)	15 082 754	12 085 219	7117 (17)	225 (26)	9.52 (0.96)	5.24 (0.61)
Zambia	4 081 068	4 517 408	7 139 736	7 339 649	57	62	0.95	NA

### Budget per child in the birth cohort

The budget per child in the birth cohort was substantially higher in countries where donor support for vaccines was included in the budget compared with those without, although only four countries included donor funding specifically for vaccines (Cote d’Ivoire, Ethiopia, Kenya and Uganda) (Table 5). For the 19 countries with a line item for vaccine supplies, average vaccine budget per child in the birth cohort without donor support was US$5.37 in 2016 and US$4.42 in 2017, ranging from US$0.23 in Ethiopia in 2017 to US$27.96 in Congo in 2016. When accounting for donor support for vaccines in Cote d’Ivoire (2017), Ethiopia (2017), Kenya (2016) and Uganda (2017), average vaccine budget per child in the birth cohort was US$11.33, US$25.49, US$21.41 and US$3.27, respectively.

**Table 5:** Joint Reporting Form reported expenditures and budget values per child in the birth cohort (values in brackets exclude on-budget donor funding)

Country	2016 JRF Government vaccine expenditures per child in birth cohort	2017 JRF Government vaccine expenditures per child in birth cohort	2016 Government vaccine budget per child in birth cohort	2017 Government vaccine budget per child in birth cohort	2016 Government immunization budget per child in birth cohort	2017 Government immunization budget per child in birth cohort	2016 difference between immunization and vaccine budget per child in birth cohort	2017 difference between immunization and vaccine budget per child in birth cohort
Angola	26.18	17.83	NA	NA	10.93	17.66	NA	NA
Benin	3.98	4.67	NA	NA	6.33	6.34	NA	NA
Burkina Faso	6.10	8.25	2.33	2.29	3.04	2.94	0.71	0.65
Burundi	1.14	2.21	1.58	1.53	1.60	1.54	0.01	0.01
Cameroon	5.08	6.01	NA	NA	NA	6.53	NA	NA
CAR	0.75	0.70	2.74	2.94	67.51 (9.68)	23.43 (8.25)	64.76 (6.94)	20.48 (5.30)
Comoros	0.46	0.66	NA	NA	1.72	NA	NA	NA
Congo	5.88	2.33	27.96	2.87	30.04	15.89 (10.93)	2.08	13.02 (8.06)
Côte d'Ivoire	8.50	9.08	8.28 (4.29)	11.33 (7.33)	9.51 (5.51)	12.55 (8.56)	1.23 (1.23)	1.23 (1.23)
DRC	1.13	NA	1.79	0.64	4.04	1.37	2.25	0.73
Ethiopia	3.63	3.71	NA	25.49 (0.23)	NA	30.01 (2.92)	NA	4.31 (2.69)
Gambia	10.94	10.79	6.78	6.28	6.78	6.47	0.00	0.19
Guinea	0.74	0.43	6.40	4.88	6.46	11.33	0.05	6.45
Kenya	5.15	5.09	21.41 (4.56)	4.51	21.54 (4.69)	4.65	0.13 (0.13)	0.15
Lesotho	7.42	5.73	2.66	2.03	14.39	14.38	11.73	12.35
Liberia	1.86	1.12	4.09	2.96	4.09	2.96	NA	NA
Madagascar	1.50	2.93	NA	NA	1.57 (0.05)	35.09 (3.88)	NA	NA
Mali	4.13	5.44	7.39	7.19	7.84	7.59	0.45	0.41
Mauritania	4.10	3.69	2.70	5.62	3.19	6.11	0.48	0.49
Mozambique	4.26	4.41	NA	NA	2.1331 (1.45)	5.93 (0.00)	NA	NA
Niger	3.54	2.62	5.18	4.43	6.59	5.72	1.41	1.29
Nigeria	16.58	4.84	0.67	3.43	7.02	5.65	6.35	2.22
Rwanda	3.61	4.23	NA	NA	10.83 (4.48)	9.26 (5.15)	NA	NA
STP	3.52	4.36	NA	NA	4.13	4.05	NA	NA
Senegal	3.38	3.65	NA	NA	16.19 (8.23)	11.37	NA	NA
Sierra Leone	1.66	1.43	NA	NA	2.35	1.99	NA	NA
Togo	1.72	2.15	3.91	3.94	4.56	4.27	0.65	0.33
Uganda	2.53	2.88	1.33	3.27 (1.63)	14.69 (1.48)	15.21 (1.77)	13.35 (0.14)	11.94 (0.14)
Zambia	3.07	3.09	5.86	6.33	6.44	6.97	0.58	0.64

Across the 29 countries, the average Government immunization budget per child in the birth cohort was US$6.37 and US$6.25 in 2016 and 2017, respectively, varying from US$0.05 in Madagascar in 2016 to US$30.04 in Congo in 2016 (Table 5). Donor support for immunization was included in the budgets of 10 countries and the values changed substantially when this was incorporated, ranging from US$1.57 in Madagascar (2016) to US$67.51 in CAR (2016). Mozambique’s 2017 budget only included funding from Gavi, with no amounts budgeted in line items that did not refer to Gavi. Hence, it seemed as if no domestic financing was budgeted.

On average, excluding donor funds, the immunization budget comprised 1.81% and 1.98% of the total Government health budget in 2016 and 2017, respectively. This varied between 0.04% in Madagascar to 5.67% in Benin in 2016 (Table 4). When including donor funding the values seemed unrealistically high for some countries, such as 19.52% in CAR and 25.56% in Madagascar.

Without accounting for donor support, differences between the immunization and vaccine budget per child in the birth cohort varied substantially across the 19 countries where these values were available, from US$0.01 in Burundi to US$12.35 in Lesotho (Table 5). When donor support was included, the differences were even greater, such as US$64.76 per child in the birth cohort in CAR (2016). Nine of the 18 countries budgeted less than US$1 per child in the birth cohort for immunization delivery, but these were all countries that did not include on-budget donor support. Without donor support, Lesotho, Congo Republic, CAR and Guinea were outliers with delivery budgets of US$12.35, US$8.06, US$6.45 and US$5.30 per child in 2017, respectively. The relatively large budget in Lesotho was especially explained by; ‘Purchases or Production of Materials’ and ‘Subsistence (Local)’. Congo, CAR and Guinea each had one line item that dominated the 2017 immunization budget; ‘Expanded Programme on Immunization’ in Congo, ‘Internal Financing’ in CAR and ‘Hospital Building’ in Guinea. Nigeria was an outlier in 2016 due to a large budget for polio campaigns.

There was no correlation between the number of line items and immunization budget per child in the birth cohort. This was apparent from scatter plots as well as insignificant Spearman’s correlation coefficients (Supplementary Annex S3).

### Budget execution

Budget execution reports that identified spending on immunization line items could only be obtained from 8 of the 33 countries and only two countries for both 2016 and 2017 (Cote d’Ivoire and Senegal). For four of the eight countries (Burkina Faso, Mali, Niger and Senegal), execution was obtained from the BOOST database. Cote d’Ivoire, Liberia and Mozambique reported previous year’s execution in the following year’s budget showing budget execution rates for each immunization line item and allowing comparison between previous year’s execution and current funding allocations. Rwanda provided a budget execution report, but instead of the 14 line items in the 2016 budget, there was only one line for immunization in the execution report.

Budget execution was 53% in Mozambique (2016), 32% in Niger (2016), 40% in Rwanda (2016), 48% in Burkina Faso (2015), 63% in Liberia (2016/17), 98% in Mali (2016), 98% in Senegal (2016 and 2017) and 123% and 99% in Cote d’Ivoire in 2016 and 2017, respectively. The low executions in Mozambique, Niger and Burkina Faso were all due to underspends in vaccine procurement. As Rwanda only reported one line item for immunization execution, it was not possible to explain the low rate.

Comparison between executed vaccine budgets and JRF vaccine expenditures could be made for five countries: Burkina Faso, Cote d’Ivoire, Liberia, Niger and Senegal. Details are included in Supplementary Annex S4. While the differences narrowed in Cote d’Ivoire, Liberia and Niger compared with the comparison with budget allocations, the discrepancies were still 19%, 39% and 41%, respectively. The difference was wider in Burkina Faso where US$2.7 million of vaccine expenses were reported to the 2015 JRF, but the executed amount was only US$845 000. Since Senegal had a 100% execution rate there was no change in the JRF comparison, with differences of 98% and 170% in 2016 and 2017, respectively. Budget execution of donor funding in Senegal’s 2016 budget in a line item called ‘Subventions’ of US$4.4 million was not reported on in the BOOST budget execution file. Hence, this is excluded from the budget execution calculation.

When comparing immunization budget executions with JRF immunization expenditures, the differences narrowed in Cote d’Ivoire and Rwanda compared with the budgeted amounts (Supplementary Annex S4). While there was a 22% difference between the Cote d’Ivoire 2016 budget and the JRF value, there was almost complete alignment between the executed amount and the value reported to JRF. However, the differences widened in Burkina Faso, Liberia, Mali, Mozambique and Niger. Due to good budget execution in Senegal, the differences were the same as when comparing with the budget.

### Inclusion of donor funding in the budget

Burkina Faso was not included in the donor funding analysis as we only had access to budget data from BOOST and not the full document. Out of the 28 remaining countries with immunization line items, 10 (36%) incorporated amounts funded from external sources within the immunization part of the budget (Table 1). However, only CAR, Madagascar, Mozambique, Rwanda and Uganda did this for both of the years (Table 4) and only four countries (Côte d’Ivoire, Ethiopia, Kenya and Uganda) identified donor funding for vaccine purchases, in spite of all the countries receiving support for vaccine purchase from Gavi. In six of the budgets, external partners were stated by name; Congo recorded the WHO as funding source for a polio campaign, Ethiopia listed Gavi vaccine support, Kenya included Gavi in the 2016/17 development budget for immunization, but not in its 2017/18 programme budgeting budget, Madagascar gave detailed information on WHO, UNICEF and Gavi contributions, Mozambique included Gavi in a bracket in almost all line items and Uganda included line items that specified both Gavi vaccine and health systems strengthening support. In the remaining four countries (CAR, Côte d’Ivoire, Rwanda and Senegal) external financing was listed, but the source not mentioned. External funding sources were listed by name somewhere in the budget in 17 of the 32 countries (53%), but Gavi was only mentioned in eight (29%).

### Budget classification structure

There was considerable variety in the manner budgets were presented. Some budgets included all immunization line items under a common subheading, such as ‘Expanded Programme on Immunization’, while others incorporated immunization line items in several different places within the MOH budget. Another difference was that immunization line items were included in both the development and the recurrent budget in some of the countries, while others only included these in the recurrent budget. Eight of the budgets were classified as following a programme structure and the remaining 25 as input based (Table 1).

## Discussion

Immunization budget line items were identified for 29 of the 33 countries. Ghana, Malawi, Tanzania and Zimbabwe did not have budget line items dedicated to vaccines or immunization services. One of the features of programme budgeting is to reduce line item controls to avoid limits imposed by Parliament or Ministry of Finance on the amounts ministries can spend on specific types of inputs (Robinson, 2014). It can thus be expected that immunization line items may be eliminated when a country transitions from line item to programme budgeting. While internal public financial management information systems would likely specify vaccine procurement and other immunization expenses within all budget structures, these are not publicly accessible. Hence, with programme budgeting, there is a risk that visibility and transparency of government support for immunization may be reduced. This was, however, not the case with the programme budgets in Angola, Cameroon, Rwanda and Uganda. Here, immunization was a subprogramme, such as within ‘Disease Prevention and Control’ in Rwanda and ‘National Disease Control’ in Uganda. These four countries had between 5 and 17 line items for immunization in spite of using a programme structure.

Only 55% of the JRF responses to the question on existence of a line item for vaccine purchases matched the details of the budget documents. It is thus apparent that this question is either misunderstood or misinterpreted. The are several reasons the question may lead to alternative interpretations. Firstly, a country may be confused over the definition of a line item for vaccine purchases, if e.g. they only have one line item for the whole immunization programme, or if they report vaccine purchases under a non-specific title such as ‘Medical and agricultural supplies’ to ensure consistency among all budgetary programmes. Secondly, some countries may be referring to a budget line within their Integrated Financial Management Information System (IFMIS) and not in their published budget documents. Thirdly, the budget could have had a line item in previous years, which has since been removed due to budget restructuring, and the immunization programme staff are not aware. Based on our analysis, we recommend that the formulation of the question or its explanation be clarified. The purpose of the question should be considered in the context of the growing uptake of programme budgeting and sophisticated IFMIS to clarify if it is more important to have visibility and transparency of a vaccine line item in published documents, or the ability to accurately track allocations and spending. This will guide the best approach to address the first two potential issues. The third issue relates to internal government budget processes and can best be solved by building the public finance capacity of immunization staff.

Excluding the four countries with no line items, the number of immunization line items in 2017 ranged from one in three countries to 42 in Madagascar, with an average of 11.3. While many line items may increase transparency and accountability, it gives less autonomy to programme management and may constrain budget execution if there is a need to seek approval to reallocate funds between line items during implementation. Comparative country case studies are needed to be able to conclude on relative advantages and disadvantages of few versus many line items. With more countries moving to programme budgeting, there would be value in examining emerging country models and approaches to budgeting for immunization, including use of vaccination rates as a performance indicator, immunization as a subprogramme, and planning and budgeting for immunization in a programme budget where there is no reported line item. Finally, given few countries included line items for immunization-specific activities, such as campaigns and surveillance, there would be value in comparing the distinct ways countries plan and budget for such activities across different budget structures.

We found considerable differences between budget values and expenditures reported to WHO and UNICEF. While budget execution data were only obtained for eight countries, six of these had great differences between government budget execution figures and expenditures reported to WHO and UNICEF. Cote d’Ivoire and Rwanda were the best example of good alignment between budget execution and the JRF, with 2016 budget executions for immunization matching reported figures in the JRF. For vaccines, differences between budgeted allocations and reported expenditure were >50% in 84% of countries, with variations in both directions. Where the budget exceeded the value reported to the JRF, it may be explained by low budget execution. However, in three countries, the values reported to the JRF were greater than the budgeted amount. Similarly, for immunization, the difference between budget allocations and reported expenditure exceeded 50% in 66% of the countries. According to the JRF guidelines for reporting on immunization expenditures, figures should only include immunization-specific expenditures, such as vaccines, cold chain maintenance, social mobilization and immunization-specific training, and not shared systems costs (World Health Organization and UNICEF, 2015). The required expenditure types were included in most budgets, especially in countries with many immunization line items. Hence, omission of certain expenditure types is not a likely explanation for the large differences. The JRF guidelines also stipulate that on-budget donor support should be included. We found that disparities between the budget values and the JRF reported expenditures were even greater when donor funding were included. This could suggest that stakeholders have not realized that on-budget donor funding should be included in the JRF. 

A 2014 survey on how JRF financing indicators were collected and reported in 36 Gavi eligible countries found that ∼60% of countries used MOH expense records and 40% used MOH budgets as the source for vaccine and immunization expenditures (World Health Organization, 2014). Other data sources were immunization expense records and budget documents (it is not clear from the survey in what way these records are different from MOH documents). It is concerning that our study, which was based on review of MOH documents, showed wide discrepancies given the survey reported MOH budgets were a key data source.

The JRF asks for hundreds of different data points in 10 distinct areas of immunization, including vaccination coverage and reported cases of vaccine preventable disease. Stakeholders with JRF data experience have stated concerns that the expenditure data questions may not be prioritized, particularly given JRF forms are often completed by MOH staff who may not be involved in budgeting and financial operations or have public financial management experience. This was confirmed by the 2014 survey, which found country stakeholders did not understand the indicators and instructions well, had difficulties accessing expenditure information, and had problems with a non-standardized reporting process (World Health Organization, 2014). Recommendations by respondents for improving the JRF data included needs for increased technical assistance and guidance from WHO and UNICEF. In a review of vaccine resource tracking systems, Leach-Kemon *et al.* (2014) similarly concluded that ‘establishing improved feedback loops and verification mechanisms that connect country-level administrators and the international organization that support reporting efforts would enhance data quality’. Some of these recommendations have since been implemented, such as a guidance document published in 2015 (World Health Organization and UNICEF, 2015) and training in the financing indicators at regional immunization workshops. It is, however, necessary that the ambitions of data collection for the JRF reflect available resources to ensure its use and quality.

The JRF data are the only source available on immunization resources routinely collected from around 155 countries. The data are used for tracking global progress on financial sustainability, including monitoring the Global Vaccine Action Plan (World Health Organization, 2013). However, the 2014 survey found that JRF financing data are almost never used in countries (World Health Organization, 2014). This may contribute to data unreliability, as there are limited incentives for countries to report accurately.

Lydon *et al.* (2008) concluded that existence of a vaccine line item was associated with increased government budget allocations for vaccines. They showed that 86% of countries reporting to the JRF in 2006 had a vaccine line item in their national budget—up from 81% in 2000. Given JRF reported vaccine expenditures also increased during this time period, they concluded there was a positive association (Lydon *et al.*, 2008). Our study does not support the findings of Lydon *et al* and raises concerns about conclusions that rely on JRF data. We found that 44% of the countries in our sample did not have a vaccine line item despite reporting positively on this to the JRF. This included four countries with no immunization line items, and a further 10 countries where it was not possible to clearly identify a vaccine line item.

The last monitoring report of the Paris declaration on Aid Effectiveness from 2011 showed that less than half of all donor aid used countries’ Public Financial Management systems (OECD, 2012). We confirmed this finding, with external funding reported in only 31% of the countries and not consistently in both years, and Gavi only reported in 29%. Given all countries in our sample were Gavi eligible, the limited reporting of on-budget support indicates that use of country systems is not widespread. To our knowledge, there are no recommended methods on how best to include and present donor commitments in national budgets and we found diverse reporting approaches in details provided. Of the budgets reviewed, Uganda incorporated donor funding in the clearest manner. In this budget, externally funded projects were listed in three places; first in a summary stating whether the donor project was included in the recurrent or development budget, secondly in a list of donor projects with forecast disbursements over 5 years, and thirdly, all subprogramme budgets had separate columns for Government of Uganda and external financing (Uganda Ministry of Finance Planning and Economic Development, 2017).

Inconsistency in inclusion of donor funding is an important explanation for the great variation in immunization budgets per child in the birth cohort. Madagascar’s 2017 budget with detailed donor commitments showed that budget per child in the birth cohort was US$35 with donor funding and only US$3.9 without external financing. Most of the countries, including the 18 countries that did not report on donor funding, have unrealistically low immunization budget allocations per child in the birth cohort, such as US$1.54 in Burundi, US$1.99 in Sierra Leone and 2.96 in Liberia. Since vaccine costs of the ‘standard’ childhood vaccination schedule amounts to at least US$20 per child in the birth cohort, the budget allocations do not align with the amount of funding known to be spent on immunization (Ahanhanzo *et al.*, 2015; Griffiths *et al.*, 2016; Geng *et al.*, 2017). Explanations for the relatively low budget allocations include omission of donor funding, exclusion of ‘shared costs’, which may be reported elsewhere in the budget, and inaccurate budget predictions. The hypothesis of inaccurate budget estimates is supported by relatively wide variations between the 2016 and 2017 budgets for some countries.

The 2017 Open Budget Survey concluded that budget transparency is inadequate in most countries. After 10 years of steady progress, the 2017 survey showed a modest decline in average global budget transparency scores, from 45 in 2015 to 43 in 2017 for the 102 countries surveyed in both rounds (scores are out of a possible 100) (International Budget Partnership, 2017). Low budget transparency was confirmed by our study with only 19 of the 33 MOH budgets available online. In five countries of our sample, it was not possible to obtain budget documents through in country requests from government (Chad, Eritrea, Guinea Bissau, Somalia and South Sudan). These are all fragile countries where there are additional challenges to budget transparency. The two online budget portals, BOOST and CABRI, are extremely valuable for improving budget transparency. We did, however, find that sector budgets were not systematically included in the databases.

The greatest hindrance for budget transparency is the lack of budget execution reports. We were only able to access these in 8 out of 33 countries, four of which were obtained from the BOOST database. Trust in government budgets is weak in many countries. Stakeholders have reported that some spending departments do not have confidence that they will receive the funds that have been allocated in the budget process, and some community groups have limited confidence that government revenues are being well used (International Budget Partnership, 2017). Public presentation of budget execution reports would provide positive incentives to strengthen budget credibility and increase confidence in government budgets.

## Supplementary data

Supplementary data are available at *Health Policy and Planning* online.

## Supplementary Material

czaa040_supplementary_data
