# Supplementary material for: Budget line items for immunization in 33 African countries
Source: Health Policy Plan. 2020 May 27;35(7):753–64. doi: 10.1093/heapol/czaa040 (PMC7487328; doi:10.1093/heapol/czaa040)
Supplement: czaa040_supplementary_data [file czaa040_supplementary_data.zip › czaa040_Suppl_Data/Line item paper annex_3rd revision.docx]

**Annex to paper on ‘Budget line items for immunization in 33 African countries’**

Ulla K Griffiths^1^, Jennifer Asman^2^, Alex Adjagba^1,3^, Marina Yo^4^, James O Oguta^3^, Chloe Cho^5^

1. Health Section, UNICEF, 3 UN Plaza, New York, NY 10017, USA
2. Social Policy Section, UNICEF, 3 UN Plaza, New York, NY 10017, USA
3. Health Section, UNICEF, Eastern and Southern Africa Regional Office, PO Box 44145, 00100 Nairobi, Kenya
4. Health Section, UNICEF, Western and Central Africa Regional Office, P.O. Box 29720, Yoff Dakar, Senegal
5. International Budget Partnership, 750 First Street NE, Suite 700, Washington, D.C. 20002

**Contents**

[Annex 1: Definitions of Government expenditures in the Joint Reporting Form 2](#_Toc38638321)

[Annex 2: Budget documents used in the analysis 3](#_Toc38638322)

[Annex 3: Correlation between number of line items and immunization budget per child in the birth cohort 6](#_Toc38638323)

[Annex 4: Comparison between budget execution data and JRF expenditure data in countries with execution data available 8](#_Toc38638324)

# **Annex 1: Definitions of Government expenditures in the Joint Reporting Form**

In 2015, the WHO, UNICEF and the Gavi Secretariat issued guidance on the data that should be included when reporting on the financing indicators in the Joint Reporting Form (JRF). The guidance can be found here:

<https://www.who.int/immunization/programmes_systems/financing/data_indicators/JRF_guidance_note.pdf>

For vaccines, the guidance defines Government expenditures as:

*Government expenditures include all funds allocated through the national and subnational government budgets, social health insurance and pooled financing. The term “vaccines used in routine immunization” includes expenditures on traditional, new and underused vaccines. It also includes government financing of the Gavi co-financing payments. Excluded from this indicator are vaccine expenditures for supplemental immunization activities (SIAs), extra-budgetary financing from donors, and out-of-pocket and informal private expenditures.*

For immunization, government expenditures are defined as:

*This figure should include recurrent immunization-specific expenditures for routine immunization financed by the government. It should also include expenditures for routine vaccines (traditional, new and underused) and vaccine co-financing payments using government funds, associated injection supplies, salaries and per diems of health staff working full-time on immunization, transport specific for immunization, vehicles and cold-chain maintenance, immunization-specific training, social mobilization, monitoring and surveillance, and programme management. Government expenditures include all administrative levels (national and subnational), all funds allocated through the national and subnational government budgets, social health insurance and pooled financing. Excluded from this indicator are shared health system costs, extra-budgetary financing from donors, and out-of-pocket and informal private expenditures.*

# **Annex 2: Budget documents used in the analysis**

| **Country** | **2016 budget documents** | **2017 budget documents** |
| --- | --- | --- |
| Angola | República de Angola, Ministério das Finanças, Exercicio 2016, Emissão: 05/08/2016 | Republica de Angola, Ministério das Finanças, Exercicio 2017, Emissão: 22/12/2016 |
| Benin | Budget de l’Etat Gestion 2017, Présentation Détaillée des Dépenses. 36 MINISTERE DE LA SANTE | Budget de l’Etat Gestion 2017, Présentation Détaillée des Dépenses. 36 MINISTERE DE LA SANTE |
| Burkina Faso  (2014 and 205) | BOOST. Spending and Performance. Data and Results.  http://boost.worldbank.org/country/burkina-faso | BOOST. Spending and Performance. Data and Results.  http://boost.worldbank.org/country/burkina-faso |
| Burundi | Republique du Burundi, Cabinet du President, Loi N° 1/22 du 31 Decembre 2015 portant fixation du budget general de la republique du Burundi pour l’exercice 2016 | Republique du Burundi, Cabinet du President, Loi N° 1/20 du 31 Decembre 2016 portant fixation du budget general de la republique du Burundi pour l’exercice 2017 |
| Cameroon | NA | Republic de Cameroun, Ministere de La Sante Publique |
| CAR | République Central africaine, Ministère des Finances et du Budget, Direction Générale du Budget. Budget 2016 par Chapitres | République Central africaine, Ministère des Finances et du Budget, Direction Générale du Budget. Budget 2017 par Chapitres |
| Chad | NA | NA |
| Comoros | Union de Comores, Presidént de l’Union, Decret N° 15-240/PR, Moroni le 31 Decembre 2015, Loi des Finances Exercise 2016 | NA |
| Congo | Republique du Congo, Ministere des - Finances- Budget et Portefeuille Public, Depenses D Investissement 2016, Ministere: 71 Sante et de la Population, Page 1 / 60  Section: 811 Cabinet  Republique du Congo, Ministere des - Finances- Budget et Portefeuille Public, Transferts 2016, Ministere: 71 Sante et de la Population, Page 1 / 60  Section: 811 Cabinet | Republique du Congo, Ministere des - Finances- Budget et Portefeuille Public, Depenses D Investissement 2017, Ministere: 71 Sante et de la Population, Page 1 / 60  Section: 811 Cabinet  Republique du Congo , Ministere des - Finances- Budget et Portefeuille Public, Transferts 2017, Ministere: 71 Sante et de la Population, Page 1 / 60  Section: 811 Cabinet |
| Côte d'Ivoire | Republique de Cote d’Ivoire, Ministere Aupres du Premier Ministre charge du Budget, Loi de Finances portant budget de ‘Etat pour l’Annee 2016  Direction Générale du Budget et des Fiances Tirage du: 03/01/17. Situation d’exécution, Section 24 Ministere de la Sante et de l’hygiene Publique | Republique de Cote d’Ivoire, Ministere Aupres du Premier Ministre charge du Budget et du Portefeuille de l’Etat, Budget 2017 |
| DRC | Republique Démoratique du Congo, Ministére du Budget, Loi du Finances N° 15/021 du 31 Decembre 2015 pour l’exercise 2016, volume II – Depenses, Kinshasa, Janvier 2016 | Republique Démoratique du Congo, Ministére du Budget, Document N° 6, Developpement par title des credits de l’exercice 2017, Kinshasa, Octobre 2016 |
| Eritrea | NA | NA |
| Ethiopia | NA | Ethiopia Federal Government 2009 Fiscal year approved capital budget |
| Gambia | Government of Gambia, Recurrent Budget detailed estimates of expenditures 2016 | Government of Gambia, Recurrent Budget detailed estimates of expenditures 2017 |
| Ghana | NA | Government of Ghana, Budget Volume - YTD March 2017, Ministry: 029 - Ministry of Health (MoH), GIFMIS Budget Module, Jan 19, 2017 time:4:56:42 PM |
| Guinea | Republique de Guinee, Ministere de l’Economie des Fiances, Budget 2016, 18 Ministére de la Santé et Hygiene Publique, Conakry, le 13 Juillet 2017 | Republique de Guinee, Ministere de l’Economie des Fiances, Budget 2017, 18 Ministére de la Santé et Hygiene Publique, Conakry, le 13 Juillet 2017 |
| Guinea Bissau | NA | NA |
| Kenya | 2016/2017 Estimates of Development Expenditure of the Government of Kenya for the year ending 30^th^ June 2017  VOLUME II, (VOTES D1065-D1132), June, 2016 | Government of Kenya, Vote D1081 Ministry of Health, Development expenditure summary 2016/17 and projected expenditure estimates for 2017/18 – 2018/19 |
| Lesotho | Government of Lesotho, MoH 2016/17, 2017/18 Capital Budget | Government of Lesotho, MoH 2016/17, 2017/18 Capital Budget |
| Liberia | The Republic of Liberia, National Budget, Fiscal Year 2017-2018, Ministry of Finance and Development Planning | The Republic of Liberia, National Budget, Fiscal Year 2018/2019, Ministry of Finance and Development Planning |
| Madagascar | Repoblikan’i Madagasikara,  Fitiavana-Tanindrazana-Fandrosoana, Annexe de la Loi N° 2015 -050, Portant Loi de Finances pour 2016, Budget d’execution, Ministere de la Sante Publique, Decembre 2015 | Repoblikan’i Madagasikara,  Fitiavana-Tanindrazana-Fandrosoana, Annexe de la Loi N° 2016 -032 du 28 Decembre 2016, Portant Loi de Finances pour 2017, Budget d’execution, Ministere de la Sante Publique, Decembre 2016 |
| Malawi | Government of Malawi, Table 5: 2016/17 Recurrent and Development Expenditure as at 31 June 2017 | Government of Malawi, Draft Estimates of Expenditure on Recurrent and Capital Budget for the Financial Year 2017/18. Program Based Budget, Budget Document No. 5 |
| Mali | Republique du Mali, Ministere de la Sante & Hygiene Publique, Situation d’execition des depenses de l’exercice 2016 a la date du 31/12/2016 | Republique du Mali, Ministere de l’economie et des finances direction generale du budget, C.B.M.T 2018-2020 |
| Mauritania | Republique Islamique de Mauritanie, Loi de Finances Budget Fonctionnement 2016 (Définitive), 53 – Ministère de la Santé | Republique Islamique de Mauritanie, Loi de Finances Budget Fonctionnement 2017 (Définitive), 53 – Ministère de la Santé |
| Mozambique | República De Moçambique, Relatório de Execução do Orçamento Do Estado  Ano 2017 Janeiro a Dezembro | República De Moçambique, Relatório de Execução do Orçamento Do Estado  Ano 2017 Janeiro a Dezembro |
| Niger | Republique du Niger, Journal Officiel, 31 décembre 2015, Special N° 36, 82^éme^ Année | Republique du Niger, Journal Officiel, 31 décembre 2016, Special N° 36, 83^éme^ Année |
| Nigeria | Federal Republic of Nigeria, 2016 Appropriation Act | Federal Republic of Nigeria, 2017 Appropriation Act |
| Rwanda | Official Gazette n°17 of 24/04/2017  2015-2016 Budget Execution by Programs | Official Gazette n° Special of 30/06/2017 |
| Sao Tome | República Democratica De São Tomé E Principe Ministério Das Finanças,  Comércio E Economia Azul Direcção Do Orçamento, Resumo De Despesas Por Órgão E Fonte De Recurso, Exercício 2016 | República Democratica De São Tomé E Principe Ministério Das Finanças,  Comércio E Economia Azul Direcção Do Orçamento, Resumo de Despesas por u.g.-funçãosf-pg-p/a-fr-ac-Natureza Económica, Exercício 2017 |
| Senegal | Republique du Senegal, Tableau Recapitulatif Par Titre, Chapitre, Article, Paragraphe Et Ligne, Section: 54 Ministère de la Santé et de l'Action Sociale, Loi de Finances pour l'année 2016 | BOOST |
| Sierra Leone | Government of Sierra Leone  Annex 1–Budget Profile for Fy 2015–2019 | Government of Sierra Leone  Annex 1–Budget Profile for Fy 2015–2019 |
| Tanzania | Government of Tanzania, Vote 52 Ministry of Health and Social Welfare, 2015/16 estimates | VOTE 052, Ministry of Health, Community Development, Gender, Elderly And Children – Health, 2018/19 |
| Somalia | NA | NA |
| South Sudan | NA | NA |
| Togo | Budget de l’Etat, Gestion 2016 Depenses, section 610 Santé et Protection Sociale | Budget de l’Etat, Gestion 2017 Depenses, section 610 Assemblée Nationale |
| Uganda | The Republic of Uganda, Draft Estimates of Revenue and Expenditure (Recurrent and Development), FY 2017/18  VOLUME I: Central Government Votes  For the Year Ending on the 30th June 2018 | The Republic of Uganda, Draft Estimates of Revenue and Expenditure (Recurrent and Development), FY 2017/18  VOLUME I: Central Government Votes  For the Year Ending on the 30th June 2018 |
| Zambia | Activity based annual budget, Ministry of Health – Mother and Child Health. 2016 | Activity based annual budget, Ministry of Health – Mother and Child Health. 2017 |
| Zimbabwe | Zimbabwe Budget Estimates for the year ending December 31, 2017. Presented to Parliament by the Minister of Finance and Economic Development on Thursday December 8, 2016 | Zimbabwe Budget Estimates for the year ending December 31, 2017. Presented to Parliament by the Minister of Finance and Economic Development on Thursday December 8, 2016 |

# **Annex 3: Correlation between number of line items and immunization budget per child in the birth cohort**

**Figure A1: Correlation between number of line items and immunization budget (2016)**

**Figure A2: Correlation between number of line items and immunization budget (2017)**

**Spearman's correlation coefficients to assess relationship between number of line items and immunization budget per child in the birth cohort**

**2016**

Number of obs = 27

Spearman's rho = -0.0098

Test of Ho: budget16 and lineitems16 are independent

Prob > |t| = 0.9613

**2017**

Number of obs = 28

Spearman's rho = 0.2111

Test of Ho: budget17 and lineitems17 are independent

Prob > |t| = 0.2808

# **Annex 4: Comparison between budget execution data and JRF expenditure data in countries with execution data available**

**Table A1: Vaccine budget execution compared to vaccine expenditures reported to the Joint Reporting Form**

| **Country** | **2016 government vaccine budget**  **(US$)** | **2017 government vaccine budget**  **(US$)** | **2016 government vaccine budget executed (US$)** | **2017 government vaccine budget executed**  **(US$)** | **2016 JRF vaccine expenditures (US$)** | **2017 JRF vaccine expenditures (US$)** | **2016 budget execution as % of JRF** | **2017 budget execution as**  **% of JRF** |
| --- | --- | --- | --- | --- | --- | --- | --- | --- |
| Burkina Faso | 1,690,761 | 1,690,761 | NA | 845,381 | 4,436,512 | 6,095,849 | NA | 14% |
| Côte d'Ivoire | 7,235,725 | 10,070,447 | 8,860,272 | NA | 7,423,912 | 8,069,068 | 119% | NA |
| Liberia | 650,000 | 477,404 | 412,201 | NA | 296,050 | 180,500 | 139% | NA |
| Niger | 5,171,129 | 4,567,165 | 2,070,923 | NA | 3,534,761 | 2,696,731 | 59% | NA |
| Senegal | 3,671,113 | 5,457,876 | 3,671,113 | 5,457,876 | 1,854,949 | 2,021,879 | 198% | 270% |

**Table A2: Immunization budget execution compared to immunization expenditures reported to the Joint Reporting Form (values in bracket excludes on-budget donor funding)**

| **Country** | **2016 government immunization budget** | **2017 government immunization budget** | **2016 government immunization budget executed** | **2017 government immunization budget executed** | **2016 JRF vaccine expenditures** | **2017 JRF vaccine expenditures** | **2016 budget execution as % of JRF** | **2017 budget execution as % of JRF** |
| --- | --- | --- | --- | --- | --- | --- | --- | --- |
| Burkina Faso | 2,207,123 | 2,169,113 | 1,048,273 | NA | 6,406,923 | 8,949,353 | 16% | NA |
| Côte d'Ivoire | 8,307,265 | 11,162,078 | 10,193,119 | 11,042,164 | 10,130,041 | 10,022,706 | 101% | 110% |
| Liberia | 650,000 | 477,404 | 412,201 | NA | 758,936 | 858,500 | 54% | NA |
| Mali | 6,036,313 | 5,942,526 | 5,907,701 | NA | 16,984,040 | 7,405,244 | 35% | NA |
| Mozambique | 2,394,196  (2,012,791) | 6,801,072  (0.00) | 1,229,236  (1,265,426) | NA | 5,851,418 | 6,062,517 | 53%  (61%) | NA |
| Niger | 6,581,286 | 5,898,754 | 2,078,144 | NA | 4,188,796 | 4,236,769 | 50% | NA |
| Rwanda | 4,019,255 (1,662,740) | 3,442,586 (1,916,460) | 1,589,620 | NA | 1,618,699 | 1,797,713 | 98% | NA |
| Senegal* | 8,881,861  (4,516,633) | 6,304,005 | (4,235,544) | 6,176,334 | 3,158,583 | 10,176,155 | 134% | 61% |

* Execution of donor funding is missing from budget execution in Senegal BOOST file
