# Supplementary material for: Budget line items for immunization in 33 African countries
Source: Health Policy Plan. 2020 May 27;35(7):753–64. doi: 10.1093/heapol/czaa040 (PMC7487328; doi:10.1093/heapol/czaa040)
Supplement: czaa040_supplementary_data [file czaa040_supplementary_data.zip › czaa040_Suppl_Data/Table 1_line item paper_2nd revision.docx]

**Table 1: Number of immunization line items in Ministry of Health budget**

| **Country** | **Online source for MOH budget** | **Number of line items 2016** | **Number of line items 2017** | **Budget execution available** | **Donor funding incorporated in budget** | **Budget structure** |
| --- | --- | --- | --- | --- | --- | --- |
| Angola | NA | 5 | 5 | No | No | Programme |
| Benin | NA | 8 | 8 | No | No | Input based |
| Burkina Faso* | BOOST | 29 | 29 | Yes | No | Input based |
| Burundi | Gov. website, BOOST | 3 | 3 | No | No | Input based |
| Cameroon | NA | NA | 13 | No | No | Programme |
| CAR | CABRI | 16 | 16 | No | Yes | Input based |
| Comoros | Gov. website | 1 | NA | No | No | Input based |
| Congo | NA | 5 | 5 | No | Yes | Input based |
| Côte d'Ivoire | NA | 30 | 31 | Yes | Yes | Input based |
| DRC | NA | 8 | 8 | No | No | Input based |
| Ethiopia | NA | NA | 9 | No | Yes | Input based |
| Gambia | NA | 1 | 6 | No | No | Input based |
| Ghana | Gov. website | 3 | 0 | No | No | Programme |
| Guinea | NA | 14 | 17 | No | No | Input based |
| Kenya | BOOST, CABRI, Gov. website | 11 | 10 | No | Yes | Input based |
| Lesotho | NA | 6 | 11 | No | No | Input based |
| Liberia | CABRI | 2 | 2 | Yes | No | Input based |
| Madagascar | NA | 17 | 42 | No | Yes | Input based |
| Malawi | Gov. website | 0 | 0 | No | No | Programme |
| Mali | BOOST | 6 | 6 | Yes | No | Input based |
| Mauritania | BOOST | 6 | 10 | No | No | Input based |
| Mozambique | NA | 25 | 30 | Yes | Yes | Input based |
| Niger | Gov. website, BOOST | 10 | 10 | Yes | No | Input based |
| Nigeria | Gov. website | 6 | 9 | No | NA | Input based |
| Rwanda | CABRI | 14 | 8 | Yes | Yes | Programme |
| Sao Tome | CABRI | 1 | 1 | No | No | Input based |
| Senegal | BOOST | 13 | 14 | Yes | Yes | Input based |
| Sierra Leone | Gov. website | 1 | 1 | No | No | Input based |
| Tanzania | NA | 0 | 0 | No | No | Programme |
| Togo | BOOST | 2 | 2 | No | No | Input based |
| Uganda | BOOST, CABRI, Gov. website | 17 | 17 | No | Yes | Programme |
| Zambia | CABRI | 4 | 4 | No | No | Input based |
| Zimbabwe | NA | NA | 0 | No | No | Programme |

*Budgets for Burkina Faso are for 2014 and 2015.

CAR: Central African Republic, DRC: Democratic Republic of Congo, NA: Not available
