# Supplementary material for: Budget line items for immunization in 33 African countries
Source: Health Policy Plan. 2020 May 27;35(7):753–64. doi: 10.1093/heapol/czaa040 (PMC7487328; doi:10.1093/heapol/czaa040)
Supplement: czaa040_supplementary_data [file czaa040_supplementary_data.zip › czaa040_Suppl_Data/Table 2_line item paper_2nd revision.docx]

**Table 2: Number and types of line items in immunization budgets (most recent year available)**

| **Country** | **Vaccine supplies** | **Gavi co-financing** | **Staff/**  **salaries/**  **allowances** | **Office supplies** | **Fuel and transport** | **Utilities** | **Mainte-**  **nance** | **Vaccination campaigns** | **Printing/**  **Child**  **Health Records** | **Surveil-**  **lance** | **Cold chain** | **Other** | **Total** |
| --- | --- | --- | --- | --- | --- | --- | --- | --- | --- | --- | --- | --- | --- |
| Angola |  |  |  |  |  |  |  |  |  |  |  | 5 | 5 |
| Benin |  |  | 7 |  |  |  |  |  |  |  |  | 1 | 8 |
| Burkina Faso | 1 |  | 12 | 1 | 3 |  | 2 |  | 1 |  |  | 9 | 29 |
| Burundi | 1 | 1 |  |  |  |  |  |  |  |  |  | 1 | 3 |
| Cameroon |  | 2 | 1 | 1 | 2 |  | 2 |  | 1 |  |  | 4 | 13 |
| CAR | 1 |  | 1 | 1 | 3 |  |  | 3 | 1 | 1 |  | 5 | 16 |
| Comoros |  |  |  |  |  |  |  |  |  |  |  | 1 | 1 |
| Congo | 1 |  |  |  |  |  |  | 2 |  | 1 |  | 1 | 5 |
| Côte d'Ivoire | 2 | 1 | 4 | 4 | 3 | 2 | 7 |  |  |  |  | 8 | 31 |
| DRC | 1 | 1 | 1 | 1 | 1 |  |  |  |  |  |  | 3 | 8 |
| Ethiopia | 1 |  | 1 | 1 | 1 |  |  |  | 1 |  |  | 4 | 9 |
| Gambia | 1 |  |  | 2 |  |  | 1 |  |  |  |  | 2 | 6 |
| Guinea | 2 |  |  | 2 | 2 | 1 | 6 |  |  |  |  | 4 | 17 |
| Kenya | 2 |  |  | 1 | 2 | 1 |  |  |  |  |  | 4 | 10 |
| Lesotho | 1 |  | 3 | 1 |  |  |  |  | 2 |  |  | 4 | 11 |
| Liberia | 2 |  |  |  |  |  |  |  |  |  |  | 0 | 2 |
| Madagascar |  |  | 3 | 7 | 9 | 9 | 4 |  | 1 | 1 |  | 8 | 42 |
| Mali | 1 |  | 1 |  | 2 |  |  |  |  |  |  | 2 | 6 |
| Mauritania | 1 |  |  | 2 |  |  | 4 |  |  |  |  | 2 | 9 |
| Mozambique |  |  |  |  |  |  |  |  |  |  |  | 30 | 30 |
| Niger | 3 | 1 |  | 2 | 1 |  |  | 1 |  |  | 1 | 1 | 10 |
| Nigeria | 3 |  |  |  |  |  |  |  |  |  |  | 6 | 9 |
| Rwanda |  |  |  | 1 | 2 | 1 |  |  |  |  |  | 4 | 8 |
| Sao Tome |  |  |  |  |  |  |  |  |  |  |  | 1 | 1 |
| Senegal |  |  |  |  | 3 |  | 1 |  |  |  |  | 10 | 14 |
| Sierra Leone |  |  |  |  |  |  |  |  |  |  |  | 1 | 1 |
| Togo | 1 |  |  |  |  |  |  |  |  |  |  | 1 | 2 |
| Uganda | 1 |  | 1 | 3 | 3 | 1 | 2 |  |  |  |  | 6 | 17 |
| Zambia | 1 |  |  |  |  |  |  |  | 1 |  | 1 | 1 | 4 |
| **Total** | **27** | **6** | **35** | **30** | **37** | **15** | **29** | **6** | **8** | **3** | **2** | **129** | **327** |
