# Supplementary material for: Budget line items for immunization in 33 African countries
Source: Health Policy Plan. 2020 May 27;35(7):753–64. doi: 10.1093/heapol/czaa040 (PMC7487328; doi:10.1093/heapol/czaa040)
Supplement: czaa040_supplementary_data [file czaa040_supplementary_data.zip › czaa040_Suppl_Data/Table 3_line item paper_2nd revision.docx]

**Table 3: Vaccine budgets compared to vaccine expenditures reported in Joint Reporting form and co-financing amounts (US$) (Figures in brackets exclude on-budget donor funding)**

| **Country** | **2016 vaccine supply budget** | **2017 vaccine supply budget** | **2016 JFR Government vaccine expenditures** | **2017 JFR Government vaccine expenditures** | **2016 vaccine budget as % of JRF** | **2017 vaccine budget as % of JRF** | **2016**  **co-financing** | **2017**  **co-financing** | **2016 co-financing as % of vaccine budget** | **2017 co-financing as % of vaccine budget** |
| --- | --- | --- | --- | --- | --- | --- | --- | --- | --- | --- |
| Burkina Faso* | 1,690,761 | 1,690,761 | 3,388,963 | 2,777,176 | 38% | 28% | 1,242,661 | 1,435,032 | 73% | 85% |
| Burundi | 707,129 | 695,042 | 510,053 | 1,003,776 | 139% | 69% | 511,140 | 494,940 | 72% | 71% |
| CAR | 455,306 | 489,611 | 124,244 | 115,961 | 366% | 422% | 167,326 | 121,950 | 37% | 25% |
| Congo | 4,974,636 | 515,380 | 1,047,203 | 419,176 | 475% | 123% | 488,566 | 746,760 | 10% | 145% |
| Côte d'Ivoire | 7,235,725  (3,746,604) | 10,070,447  (6,515,910) | 7,423,912 | 8,069,068 | 97%  (50%) | 125%  (81%) | 1,334,440 | 916,555 | 18%  (36%) | 9%  (14%) |
| DRC | 5,983,990 | 2,193,001 | 3,775,045 | NA | 159% | NA | 10,039,985 | 0 | 168% | 0% |
| Ethiopia | NA | 83,703,491  (750,062) | 11,835,511 | 12,190,577 | NA | 687% (6%) | 4,402,641 | 4,490,322 | NA | 5% (599%) |
| Gambia | 546,888 | 514,938 | 882,000 | 884,500 | 62% | 58% | 136,997 | 111,181 | 25% | 22% |
| Guinea | 2,862,661 | 2,208,705 | 328,920 | 194,511 | 870% | 1136% | 223,469 | 232,500 | 8% | 11% |
| Kenya** | 32,540,471  (6,925,810) | 6,925,810 | 7,824,000 | 7,824,000 | 416% (89%) | 89% | 2,928,157 | 592,517 | 9% (42%) | 9% |
| Lesotho | 163,159 | 124,667 | 456,019 | 352,071 | 36% | 35% | 31,583 | 75,818 | 19% | 61% |
| Liberia | 650,000 | 477,404 | 296,050 | 180,500 | 220% | 264% | 296,050 | 194,637 | 46% | 41% |
| Mali | 5,690,525 | 5,625,423 | 3,180,350 | 4,261,159 | 179% | 132% | 1,677,712 | 1,750,795 | 29% | 31% |
| Mauritania | 397,309 | 838,120 | 601,825 | 550,325 | 66% | 152% | 303,887 | 199,617 | 76% | 24% |
| Niger | 5,171,129 | 4,567,165 | 3,534,761 | 2,696,731 | 146% | 169% | 0 | 0 | 0% | 0% |
| Nigeria | 4,883,658 | 25,184,626 | 120,000,000 | 35,480,412 | 4% | 71% | 29,268,750 | 38,100,622 | 599% | 151% |
| Togo | 1,011,790 | 1,030,760 | 445,686 | 561,763 | 227% | 183% | 448,855 | 446,197 | 44% | 43% |
| Uganda | 2,339,114 | 5,847,786  (2,923,893) | 4,432,069 | 5,151,336 | 53% | 114% (57%) | 2,054,296 | 1,708,614 | 88% | 29% (58%) |
| Zambia | 3,712,603 | 4,100,461 | 1,946,125 | 2,000,617 | 191% | 205% | 1,678,997 | 1,639,856 | 45% | 40% |

*Burkina Faso values are for 2014 and 2015

**Kenya’s financial year is June-July. Co-financing is aligned with the financial year. 2016 values cover July 2016-June 2017. 2017 values cover July 2017-June 2018.

NA: Not available, CAR: Central African Republic, DRC: Democratic Republic of Congo

Source of co-financing data is UNICEF Supply Division
