# Supplementary material for: Budget line items for immunization in 33 African countries
Source: Health Policy Plan. 2020 May 27;35(7):753–64. doi: 10.1093/heapol/czaa040 (PMC7487328; doi:10.1093/heapol/czaa040)
Supplement: czaa040_supplementary_data [file czaa040_supplementary_data.zip › czaa040_Suppl_Data/Table 4_line item paper_2nd revision.docx]

**Table 4: Government immunization budgets compared to immunization expenditures reported in the Joint Reporting Form (Figures in brackets exclude on-budget donor funding)**

| **Country** | **2016 immunization budget** | **2017 immunization budget** | **2016 JFR Government immunization expenditures** | **2017 JFR Government immunization expenditures** | **Imm. budget as % of 2016 JRF x** | **Imm. budget as % of 2017 JRF** | **2016 imm. budget % of total Government health budget** | **2017 imm. budget % of total Government health budget** |
| --- | --- | --- | --- | --- | --- | --- | --- | --- |
| Angola | 13,187,664 | 21,790,617 | NA | NA | NA | NA | 0.71% | 3.85% |
| Benin | 2,554,617 | 2,599,609 | 3,951,102 | 2,653,336 | 65% | 98% | 5.67% | 3.02% |
| Burkina Faso | 2,207,123 | 2,169,113 | 3,527,388 | 3,864,493 | 34% | 24% | 0.72% | 0.51% |
| Burundi | 712,568 | 700,481 | 597,351 | 1,208,865 | 119% | 58% | 1.19% | 0.74% |
| Cameroon | NA | 5,625,765 | 4,542,974 | 5,473,014 | NA | 103% | NA | 1.57% |
| CAR | 11,198,011 (1,606,013) | 3,895,104 (1,370,911) | 124,244 | 168,359 | 9,013% (1,293%) | 2,314% (814%) | 19.52% (2.80%) | 8.97% (3.16%) |
| Comoros | 44,968 | NA | 72,289 | 90,820 | 62% | 0% | 1.26% | NA |
| Congo | 5,345,205 | 2,853,255  (1,963,365) | 1,161,872 | 453,535 | 460% | 629% (433%) | 1.71% | 1.25% (0.86%) |
| Côte d'Ivoire | 8,307,265  (4,818,144) | 11,162,078 (7,607,540) | 10,130,041 | 10,022,706 | 82% | 111% | 2.31%  (1.34%) | 1.51%  (1.03%) |
| DRC | 13,484,026 | 4,673,444 | 3,934,332 | 633,902 | 343% | 737% | 2.14% | 1.42% |
| Ethiopia | NA | 98,557,551  (9,589,559) | 49,721,033 | 51,212,664 | NA | 192% (19%) | NA | 9.61% (0.94%) |
| Gambia | 546,888 | 530,429 | 883,400 | 886,600 | 62% | 60% | NA | 3.27% |
| Guinea | 2,886,757 | 5,130,062 | 497,671 | 1,506,409 | 580% | 341% | 3.31% | 3.50% |
| Kenya | 32,738,555  (7,123,894) | 7,149,840 | 8,094,991 | NA | 404% (88%) | NA | 5.51% (1.20%) | 1.20% |
| Lesotho | 883,776 | 883,776 | 784,917 | 518,841 | 113% | 170% | 0.66% | 0.57% |
| Liberia | 650,000 | 477,404 | 758,936 | 858,500 | 86% | 56% | 0.84% | 0.62% |
| Madagascar | 1,302,628 (43,396) | 29,594,519 (3,268,698) | 1,250,038 | 2,467,250 | 104% (3%) | 119% (32%) | 1.31% (0.04%) | 25.56% (2.82%) |
| Mali | 6,036,313 | 5,942,526 | 16,984,040 | 7,405,244 | 36% | 80% | 3.03% | 2.46% |
| Mauritania | 468,467 | 910,963 | 1,089,278 | 1,857,831 | 43% | 49% | 0.55% | 0.93% |
| Mozambique | 2,394,196  (2,012,791) | 6,801,072  (0) | 5,851,418 | 6,062,517 | 41%  (34%) | 112%  (0%) | 0.90%  (0.75%) | 2.11%  (0%) |
| Niger | 6,581,286 | 5,898,754 | 4,188,796 | 4,236,769 | 157% | 139% | 4.42% | 4.54% |
| Nigeria | 50,821,961 | 41,439,457 | 185,000,000 | 78,145,947 | 27% | 53% | 5.15% | 4.17% |
| Rwanda | 4,019,255 (1,662,740) | 3,442,586 (1,916,460) | 1,618,699 | 1,797,713 | 248% (103%) | 191% (107%) | 2.81% (1.16%) | 2.78% (1.55%) |
| Sao Tome | 27,992 | 27,619 | 554,542 | 360,072 | 5% | 8% | 0.12% | 0.34% |
| Senegal | 8,881,861  (4,516,633) | 6,304,005 | 3,158,583 | 10,176,155 | 281% (143%) | 62% | 3.51% (1.78%) | 2.24% |
| Sierra Leone | 608,639 | 518,428 | 430,985 | 494,492 | 141% | 105% | 2.39% | 2.95% |
| Togo | 1,180,422 | 1,116,657 | 4,389,546 | 4,798,970 | 27% | 23% | 1.08% | NA |
| Uganda | 25,741,952 (2,590,569) | 27,220,225 (3,176,810) | 15,082,754 | 12,085,219 | 71% (17%) | 225% (26%) | 9.52% (0.96%) | 5.24%  (0.61%) |
| Zambia | 4,081,068 | 4,517,408 | 7,139,736 | 7,339,649 | 57% | 62% | 0.95% | NA |
